# Charting a Path Between Research and Practice in Alcoholism Treatment

**Published:** 2006

**Authors:** Dennis McCarty, Eldon Edmundson, Tim Hartnett

**Affiliations:** Dennis McCarty, Ph.D., is a professor, and Eldon Edmundson, Jr., Ph.D., is an associate professor, both in the Department of Public Health and Preventive Medicine, Oregon Health and Science University, Portland, Oregon. Tim Hartnett, M.S.W., M.H.A., is the executive director of CODA, Inc., Portland, Oregon

**Keywords:** health services research, alcohol dependence, research in practice, Researcher in Residence Program, treatment method, treatment research, treatment program, treatment provider, motivational interviewing

## Abstract

The journey between research and practice in alcoholism treatment is worthwhile but can be difficult because of the inherent differences between the treatment and research disciplines. This article describes how the path between research and practice can be navigated successfully, discusses the factors that influence the journey, and offers specific pharmaceutical and behavioral interventions as examples of research-based treatment approaches that can be implemented more widely.

The Hana Highway lures adventurers with a legendary combination of scenic splendor and driving danger. Drivers navigate countless hairpin turns and 56 one-lane bridges on the 52-mile stretch between Kahului and Hana along Maui’s northern coast. There is little room for error. On the left, surf pounds a rugged shoreline far below the road; black basalt presses close on the right. Intermittent squalls and swirling mountain mist moisten the roadway, limit visibility, and increase risk. The trip is exhausting and exhilarating and doesn’t get easier with practice.

The journey between research and practice in alcoholism treatment can resemble the Hana Highway—attractive yet treacherous, with unexpected hazards. Culture and language differ in the worlds of practice and research, and bidirectional translation is necessary. It helps to have guides pointing out signposts in each discipline, and the traveler must be receptive to the customs of each field. Researchers sensitive to clinical issues will learn that resources are limited in most practice settings and that simple interventions are more likely to be useful. Therapists, in turn, come to appreciate the value of standardized techniques and the benefits of data collection.

Researchers introducing “evidence-based” practices may face a host of objections from treatment providers. “Experience-based” treatment providers resent the implication that their treatments are not empirical; they point to the millions of men and women who have found stable recovery through these treatments. Clinicians are concerned that standardized practices inhibit individualized care. As a result, many researchers and clinicians who hope to travel between practice and research start but turn back, lose their way, or second-guess the destination. Success requires clearly defined objectives, constant negotiation with conflicting demands, and a willingness to enjoy the journey. With persistence, treatment programs can achieve the goal of providing research-guided services.

Applying science to policy and practice is both challenging and attractive, as is the use of clinical insights to guide research. Efficacy trials show that an effect is possible in controlled settings (e.g., that a particular alcoholism treatment approach is effective under specific conditions); effectiveness trials document that these effects are feasible in real practice settings. This article describes how the path between research and practice can be navigated successfully, discusses the factors that influence the journey, and offers specific pharmaceutical and behavioral interventions as examples of research-based treatment approaches that can be implemented more widely.

## The Challenge

Health care services in the United States often are not based on the latest scientific research and fail to meet patient needs. The Institute of Medicine (IOM) has challenged health care systems to fundamentally restructure the organization and delivery of care (IOM 2000). The gap between practice and research seems to be especially pronounced in the delivery of treatments for alcohol and other drug (AOD) use disorders. The IOM’s analysis *Bridging the Gap Between Practice and Research: Forging Partnerships with Community-Based Drug and Alcohol Treatment* (Lamb et al. 1998) found a broad disconnect and recommended that the National Institutes of Health and the Substance Abuse and Mental Health Services Administration (SAMHSA) invest in promoting the application of research to treatment for dependence on alcohol and other drugs. In response to these recommendations:

- The National Institute on Alcohol Abuse and Alcoholism (NIAAA) created a Researcher in Residence program to link researchers with treatment providers, deliver training and technical assistance, and foster the application of empirically based interventions in treatment programs (Hilton 2001).
- SAMHSA’s Center for Substance Abuse Treatment (CSAT) sponsored Practice Improvement Collaboratives to enhance service effectiveness through the application of science and to inform science about the challenges of practice (Cotter et al. 2005).
- SAMHSA, focusing on the “science to service cycle,” used its network of regional Addiction Technology Transfer Centers and the national One Sky Center (for American Indian tribes) to promote research-informed treatment services.
- The National Institute on Drug Abuse constructed the National Drug Abuse Treatment Clinical Trials Network and created partnerships between research centers and networks of community-based treatment centers in order to design and conduct clinical trials that test research-based interventions in the real world.
- The Veterans Affairs (VA) Health Care System developed a Substance Abuse Module for the Quality Enhancement Research Initiative (QUERI) to promote substance abuse intervention in primary care, foster the use of science-based practices, and improve care for patients with comorbid conditions (Finney et al. 2000).

These Federal agencies are working to catalyze the application of science to clinical practice. Observation and research, however, suggest that the journey between research and practice is easier to initiate than to complete. Research often is perceived as unresponsive to practitioner and patient needs, and research-based interventions may be too complex, too expensive for practice settings, not applicable to all patient groups, or not integrated with the larger system of care.

## A Road Map

The primary travel guide for the journey between science and service is Everett Rogers’s classic text *Diffusion of Innovations* (Rogers 2003; originally published in 1962), which summarizes research on the adoption and use of new technologies and provides a framework for understanding and investigating this process. Briefly, Rogers theorizes that decisions to adopt innovations are based on the attributes of the innovation. Innovations are more likely to be widely adopted if they are relatively simple, compatible with existing values, available to be tried on a limited basis, and offer observable results and advantages. People’s perceptions of innovations also are influenced by how they are communicated. The source of this communication is very important as well: Members of a group typically accept information on new technology more readily from colleagues who already have tried it.

Rogers’s diffusion theory has informed the development of two models designed to encourage clinicians to adopt evidence-based practices for treating AOD use disorders. Simpson (2002) offers a model of organizational change designed to support an organization in implementing behavioral therapies, and Thomas and colleagues outline variables that help an organization adopt new pharmacological therapies (Thomas et al. 2003; Thomas and McCarty 2004) (see table).

## Models for Promoting Organizational Change

Simpson’s (2002) model proposes that adopting innovations occurs in four interdependent stages—exposure to the new practice, deciding to adopt it, implementing it, and standardizing the practice (institutionalization of the change).

In the exposure stage, readiness to change (that is, a perceived need to change) and the presence of organizational resources (training, staff time, equipment, etc.) enhance an organization’s responsiveness to the new practice. The decision to use the new practice, the second stage, requires a leadership decision or organizational consensus; the leaders see the usefulness of the new method and are motivated to change. The third stage, implementation, requires decisionmakers to allocate sufficient resources and provide institutional support for the change effort; this stage includes monitoring to ensure correct use (i.e., fidelity) and further refinement of the new method. The final stage––acceptance of the practice as the standard of care––depends on the organizational climate for change and staff attributes such as education and training.

The Simpson model’s stages of change––inform, implement, adopt, and use––mimic the stages of the treatment process. This model conforms to a traditional view of organizational change.

In the Thomas model, organizational acceptance drives practitioner adoption—counselors and physicians use medication when the organization they work in provides clear support for the use of pharmacotherapy. Organizational acceptance is a function of:

- Organizational characteristics (e.g., corporate structure, size, and procedures).
- Practitioner characteristics (e.g., gender, age, training, and experience).
- System influences such as State regulations and insurance company policies on payment for pharmaceuticals.
- Features of the new medication (e.g., cost and effectiveness) (Thomas et al. 2003).

By emphasizing organizational acceptance, this model implies a strong role for corporate leadership. It assumes that adoption of new pharmacotherapies is more likely to occur through a top-down process of organizational change.

## Adoption of Pharmacotherapy

Naltrexone is an opiate-antagonist medication. Clinical trials suggest that naltrexone contributes to reductions in the frequency of drinking and severity of relapse among alcohol-dependent patients (e.g., O’Malley 1998; Volpicelli et al. 1992). Although there are occasional reports of no effects (Krystal et al. 2001), meta-analyses of clinical trials have found a modest but significant improvement in treatment outcomes (Kranzler and Van Kirk 2001; Streeton and Whelan 2001). A CSAT Treatment Improvement Protocol, moreover, provides clinical guidance on using naltrexone for alcoholism treatment (O’Malley 1998).

**Table:** Two Models of Adopting Treatment Innovations

Thomas’s model of adopting new pharmacological therapies*	Simpson’s model of adopting innovative behavioral therapies**
Organizational acceptance of new therapies is a function of: Organizational attributes, such as: Corporate structure Size Procedures Practitioner characteristics, such as: Gender Age Training Experience System influences, such as: State regulations Insurance company policies Features of the medication, such as: Cost Effectiveness **Most influential factor:** The organization’s decision to use the new therapy.	The stages of adoption and factors at work at each stage to enhance adoption: Stage 1: Exposure to the new practice Enhancing factors: Readiness to change (perceived need to change) Presence of organizational resources Stage 2: Deciding to use the new practice Enhancing factor: Leadership decision or organizational consensus Stage 3: Implementing the new practice Enhancing factors: Decisionmakers: Allocate sufficient resources Provide institutional support See that implementation is monitored to ensure fidelity to the innovation Stage 4: Standardization of the practice; acceptance Enhancing factors: Organizational climate for change Staff attributes, such as education and training **Most influential factor:** Organizational motivation to change, organizational consensus around the need for change, strong leadership.

SOURCES:

* Thomas et al. 2003; Thomas and McCarty 2004.

** Simpson 2002.

Despite the availability of training materials for professionals and consistent reports of significant reductions in alcohol use, it appears that few patients receive naltrexone during treatment. Even physicians who specialize in treating addictions are unlikely to prescribe naltrexone. Among the patients being treated by members of the American Academy of Addiction Psychiatry and the American Society of Addiction Medicine, about 1 in 7 (13 percent) were given naltrexone prescriptions (Mark et al. 2003*a*,*b*). Similar results were found in a survey of 400 private alcohol and drug treatment centers: 44 percent of the programs reported current use of naltrexone for about 13 percent of their patients who have a primary diagnosis of alcohol dependence (Roman and Johnson 2002).

**Figure:**
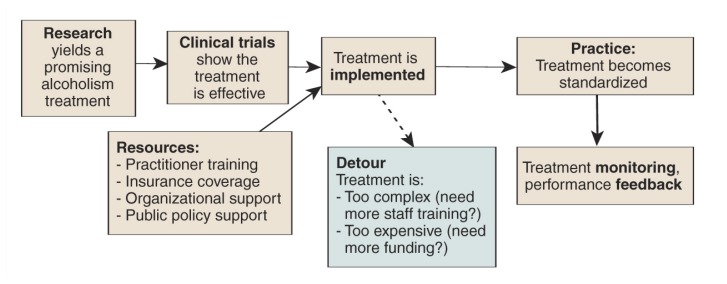
The process of translating research findings on alcoholism into new interventions and treatments.

A survey of certified addiction counselors and physicians specializing in addiction medicine also explored some barriers to naltrexone use for treating alcohol dependence (Thomas et al. 2003). Again, limited use was observed. Few counselors (5 percent) recommended naltrexone to most of their patients, and more than half (54 percent) never suggested that patients try it. A somewhat higher percentage of physicians prescribed naltrexone for patients. Eight of 10 physicians (80 percent) reported current or prior use of naltrexone with patients, but only 11 percent prescribed it “often,” and only 4 percent prescribed it for “almost all patients.”

This survey revealed factors that encouraged these professionals to use naltrexone with their patients. Organizational support was the strongest predictor of whether counselors recommended naltrexone. Patient access to insurance benefits also influenced counselor behavior. Counselors with more Medicaid patients were more likely to promote its use, naltrexone being on the Medicaid formulary in the three study States. Counselors whose patients paid for their own treatment or whose treatment was funded through State and Federal funds were less likely to recommend naltrexone to patients. Among physicians, those involved in research and those in organizations that promoted naltrexone use were more likely to prescribe the medication. Physicians in recovery were the least likely to prescribe naltrexone.

Thomas’s model suggests that an organization’s decision to use medications may be the most influential factor in the adoption process. In contrast, Simpson’s model suggests that the adoption of behavioral therapies is driven by motivation to change, an organizational consensus around the need for change, and strong leadership (Simpson 2002).

## Adoption of Behavioral Therapies

Motivational interviewing, a treatment approach that aims to increase a patient’s motivation to change, has been found effective in controlled clinical trials (Carroll et al. 2001; Miller and Rollnick 2002; Project MATCH Research Group 1997). Tests in an array of community-based AOD abuse treatment settings, moreover, suggest good potential for greater implementation of this approach. Despite encouraging research findings, however, implementing motivational interviewing in practice settings has proven to be a challenge.

The primary barrier is the complexity of the interventions. Therapeutic skills are not acquired merely from reading books, watching videos, or completing a day of training. Skill development requires practice and coaching. Thus, Simpson’s (2002) model proposes moving clinicians through a four-step process consisting of exposure (awareness and training), adoption (a commitment to try the technique), implementation (exploratory use), and routine practice (continued use). A supportive environment, appropriate supervision, and institutional supports facilitate practitioner change and promote the development of clinical skills.

This process was observed in CSAT’s Practice Improvement Collaboratives (PICs). Researchers conducting the Oregon PIC learned that implementing motivational interviewing usually required three preliminary steps: preparing the treatment agency, preparing supervisors, and finally, preparing clinicians (Dickinson et al. in press). The steps are complementary, and changing the sequence increases adoption barriers. The first step, agency preparation, includes articulating the expected outcomes––the staff and client behaviors that should follow implementation––and how motivational interviewing will help achieve those objectives. For more complex interventions, treatment programs tend to need greater preparation. The second step is supervisor preparation. Supervisors need to be well trained in order to support agency goals and therapist skill development. When the first two steps have been completed, attention can shift to counselor training and full implementation. The agency also must be prepared to sustain the intervention.

Dickinson and colleagues (in press) identified specific barriers while working with local treatment programs to implement motivational interviewing. Adherence to an evidence-based practice often requires direct or taped observation of clinical sessions, but practitioners were resistant to having their clinical sessions audiotaped for supervision and coaching. Thus, a first challenge was increasing practitioner comfort with this process. Fidelity rating was a second challenge. The clinical rating forms used in research interventions required detailed assessments of multiple facets of the therapy and therapeutic approaches, but the rating process was much too complex and time-consuming for actual practice settings. Rating forms and procedures had to be focused and simplified before fidelity could be assessed efficiently and productively. Finally, quality improvement was an ongoing challenge. Agencies seeking to promote the use of motivational interviewing had to develop forums for staff interactions and coaching that challenged staff to improve their skills. Peer review of audiotapes from clinical sessions became a useful tool in many sites and fostered a culture that supported the use of motivational interviewing.

## Reaching the Destination

Slowly, the collaboration between research and practice is influencing treatment for alcohol abuse and dependence. The road between practice and research is not an expressway; like the Hana Highway, it has many distractions and unexpected turns. Researchers and practitioners need to be wary of oncoming traffic. The Simpson and Thomas models outline some of the variables that affect the journey and begin to articulate strategies to facilitate the trip. Much remains to be learned. Practitioners, policymakers, and investigators, however, can begin to systematically manage the adventure.

In clinical settings, agency leadership sets the tone, and funding and regulations influence the practice environment. Program directors in successful agencies have a clear sense of the practices and interventions they want to use and why. They can provide clear expectations about evidence-based practices that promote their use. They prepare the supervisors, clinicians, and support staff to implement and sustain the new practices and therapies. Preparing supervisors through training and peer discussion is a critical initial investment and fosters a culture that supports the practitioners as they develop skills with new therapies. Maintaining fidelity to the intervention requires ongoing supervision; performance feedback through the use of audio- and videotaping, for example, can enhance implementation and assure adherence to the therapeutic technique.

Participating clinicians can increase their skills in training workshops if they are motivated and fairly knowledgeable; they gain the most when they receive coaching and feedback for an extended period (Miller et al. 2004). Policymakers also influence the treatment environment. Regulations, contracts, and incentives can be aligned to promote the use of research-based therapies. Because purchasing policies can have substantial impact on the adoption of new treatments, policymakers must develop financing mechanisms that promote and support the use of science-based interventions, including medications. Policymakers also can mandate the use of evidence-based practices. The Oregon General Assembly, for example, approved legislation in 2003 that requires the Oregon Mental Health and Addiction Services agency to purchase evidence-based practices. By fiscal year 2009, the State agency must demonstrate that 75 percent of its purchases are for evidence-based treatments. Other States and jurisdictions may develop similar strategies. The goal is to create strategies in which researchers, practitioners, and policymakers coordinate their efforts to support the implementation of science-based practices. Unfortunately, the roadside is littered with initiatives that were compromised by misalignment of effort or by the absence of key elements (e.g., limited use of naltrexone).

Long-term strategies, moreover, must involve changing academic and professional training programs. Many entry-level practitioners receive their primary training for addiction counseling in 2-year certificate programs offered in community colleges. The content of these programs varies considerably and may provide little introduction to behavioral and pharmacological therapies.

Like many adventures, the journey between practice and research deserves much preparation. Success requires a focused plan and attention to the factors that facilitate the adoption of desired practices. Investigators and clinicians can embrace the barriers, learn from failure, and continue to develop and nourish their skills. And, as with travel on the Hana Highway, the journey cannot be rushed. Travelers should stop frequently to observe the surroundings and appreciate their accomplishments along the way. The destination is difficult to achieve but worthwhile.
